# ClinVLA: an image-text retrieval method for promoting hospital diagnosis data analysis and patient health prediction

**DOI:** 10.3389/fphys.2025.1661960

**Published:** 2025-10-16

**Authors:** Xiao Hao, Jiaxiang Liu, Yang Chen

**Affiliations:** ^1^ School of Public Health, Henan Medical College, Zhengzhou, China; ^2^ School of Computer Science and Engineering, University of New South Wales (UNSW), Sydney, NSW, Australia; ^3^ Modern Education Technology Center, Henan Medical College, Zhengzhou, China

**Keywords:** image-text matching, health prediction, medical imaging, adapter module, deep learning

## Abstract

Medical visual-language alignment plays an important role in hospital diagnostic data analysis and patient health prediction. However, existing multimodal alignment models, such as CLIP, while performing well in some tasks, often fail to accurately capture the fine-grained alignment between complex medical images and texts, and lack the capability to handle multi-view radiological image inputs. To address these issues, this paper proposes the ClinVLA model, an efficient visual-language alignment method. Specifically, ClinVLA enhances image feature representation through an innovative multi-view input design, including both frontal and lateral views. Furthermore, ClinVLA introduces an innovative adapter module, making the model more efficient in task transfer and language transformation, significantly improving performance in cross-modal learning. Finally, by incorporating both global and local alignment losses, ClinVLA ensures semantic consistency between images and texts, optimizing the accuracy and efficiency of image-text matching. Experimental results on datasets such as CheXpert and RSNA Pneumonia show that ClinVLA improves text-to-image retrieval accuracy by over 3% compared to the best-performing similar algorithms, and increases image-to-text retrieval accuracy by approximately 5%. ClinVLA provides a new solution for medical image analysis, with broad application prospects.

## 1 Introduction

Hospital diagnostic data analysis and patient health prediction play a crucial role in medical research and clinical practice. With the rapid increase in medical data, traditional manual diagnostic methods face significant challenges (Rayed et al. 2024; Pu et al. 2024). Effectively extracting valuable information from large amounts of medical data for accurate disease prediction and early warning has become a key research focus in the field of medicine today. Medical data not only include electronic health records (EHRs), patient medical histories, and diagnostic reports but also imaging data, such as X-rays, CT scans, MRIs, etc. The integration and analysis of these data sources can provide more comprehensive support for clinical decision-making, significantly enhancing diagnostic efficiency and accuracy (Meng et al. 2024; Wu et al. 2025; Li C. et al. 2025; Cao et al. 2025).

Medical visual-language alignment, as an emerging research direction, has been widely applied to the automatic matching of medical images and texts (Shi et al. 2024; Zhang et al. 2025; Li J. et al. 2025). In this process, image data (such as radiological images) and text data (such as radiology reports and medical descriptions) are jointly analyzed through alignment techniques, enabling computers to understand the relationship between image content and corresponding reports, thereby assisting doctors in providing diagnostic recommendations Liu (2024), Ning et al. (2025). Effective image-text matching not only improves diagnostic efficiency but also reduces the risk of misdiagnosis caused by human error. This technology has broad application prospects in medical automation, telemedicine, and other fields (Xiao et al. 2024; Alsabbagh et al. 2025).

The image-text matching method based on the CLIP Radford et al. (2021) (Contrastive Language-Image Pre-training) model has been widely used in visual analysis Pham et al. (2024). CLIP pre-trains image and text encoders, mapping images and texts into the same semantic space, effectively measuring the similarity between them. For example, DeCLIP introduces deformable convolution modules to capture image details, improving image alignment accuracy; VisualBERT Li et al. (2019), Xing et al. (2025) and UNITER Chen et al. (2020) enhance the interaction between image and text features through cross-modal pre-trained models and complex attention mechanisms, thus improving matching performance; VLP (Vision-Language Pretraining) strengthens the understanding and generation of image-text relationships through large-scale visual and language datasets, especially excelling in processing complex data Tuerhong et al. (2024); CLIP-ViT combines Vision Transformer and CLIP to improve the feature extraction capability of high-resolution images, enhancing the quality of image-text matching (Zhang et al. 2024; Xiong et al. 2024; Umer and Sharif 2022).

However, CLIP-based methods still face the problem of poor visual representation ability. This is mainly reflected in their insufficient ability to process complex medical images, especially when dealing with fine-grained medical images, where traditional methods often fail to capture the detailed alignment between images and texts. Additionally, these methods usually require a large amount of pre-training data and computational resources, leading to low computational efficiency and an inability to effectively reduce the pre-training burden. More importantly, these methods often lack the ability to handle temporal multi-view radiological images, failing to fully utilize the temporal dynamic changes across multiple image acquisitions, thereby limiting their application in dynamic medical image analysis.

To address the above issues, we propose a novel method—ClinVLA (Clinical Visual-Language Alignment). This method integrates adapters and uses only 12% of the trainable parameters, significantly reducing the model’s training complexity and computational overhead. ClinVLA uses temporal multi-view radiological images as input, enhancing the visual-language alignment effect and improving the consistency of information between radiological images and radiology reports. Through this innovative design, our method not only outperforms traditional models in terms of performance but also achieves more efficient cross-modal learning, providing a new solution for medical image analysis and intelligent diagnosis.

The three contributions of this paper are as follows:

• This paper proposes an efficient image-text alignment method, ClinVLA, which enhances the semantic consistency between medical images and radiology reports through innovative adapter modules and masking modeling techniques.

• This paper adopts multi-view image input, including frontal and lateral views, which improves the alignment precision between images and texts, particularly for fine-grained medical image details.

• By introducing adapter modules, this paper significantly reduces the number of trainable parameters, improving the efficiency of task transfer and language transformation.

## 2 Related works

### 2.1 Image-text matching methods

Image-text matching has become an important research direction in the medical field. CLIP Radford et al. (2021), Kodipalli et al. (2023) performs contrastive pretraining on large-scale image-text pairs, mapping images and texts to the same semantic space, achieving good results in image-text matching tasks. Similarly, the ALIGN (A Larger-scale Image-Text Pretraining) Jia et al. (2021) method also leverages a large number of image-text pairs for joint training, further enhancing cross-modal representation capabilities. Additionally, methods such as VisualBERT Li et al. (2019) and UNITER Chen et al. (2020) introduce BERT models and utilize the bidirectional Transformer encoding ability to simultaneously process image and text features, effectively strengthening the semantic consistency between images and texts, and improving matching performance. The CLIP-ViT Wu S. et al. (2023) method combines Vision Transformer (ViT) with CLIP, providing stronger feature extraction capabilities for high-resolution images, effectively improving the performance of image-text matching at the detail level. Furthermore, T2T-ViT (Tokens-to-Token Vision Transformer) Zhao et al. (2022) introduces a Token-to-Token transformation module, improving the precision of detail capture in image feature processing and optimizing cross-modal fusion, making it particularly suitable for fine-grained image-text matching tasks. While these methods have contributed to improving the accuracy of image-text matching, they still have limitations when handling dynamic changes, temporal data, and multi-view imaging. Particularly in the medical imaging field, the temporal information between images and multi-view data often provides richer diagnostic clues, but existing models struggle to fully utilize this information.

This paper proposes the ClinVLA model, which integrates temporal multi-view images for image-text alignment. The model uses only 12% of the trainable parameters, significantly improving computational efficiency. Compared to traditional methods, ClinVLA places greater emphasis on temporal consistency between images.

### 2.2 Research progress on adapters

The adapter modules are primarily used for fast fine-tuning of pre-trained Transformer models, particularly for efficient task transfer with limited computational resources Pfeiffer et al. (2020). For example, the Adapter module inserts small networks in each layer of the Transformer model, fine-tuning only the newly added adapter parameters, which reduces computational overhead. Compacter compresses the parameter size of the adapter through low-rank decomposition, not only improving fine-tuning efficiency but also achieving better results under limited resources. AdapterFusion Pfeiffer et al. (2020) improves generalization in multi-task learning by fusing outputs from multiple adapters, allowing efficient cross-task transfer learning. The Progressive Neural Networks method, although different from traditional adapters, proposes a strategy of progressively adding new modules instead of updating the original model, which helps the model adapt to new tasks. Adapter-BERT Zhang et al. (2021) introduces adapter modules into the BERT model to reduce training costs while enabling quick adaptation to new tasks, improving efficiency in low-resource environments. These adapter modules enhance the task adaptability of Transformer models by updating only a small number of parameters while effectively controlling computational costs (Ye et al. 2023; Pfeiffer et al. 2020). However, existing adapter methods often fail to capture the fine-grained features and temporal changes in medical images, particularly when handling dynamic medical images (such as continuous radiological image sequences or images from different perspectives), where they are unable to effectively leverage the temporal and spatial relationships between images.

## 3 Methods

**FIGURE 1:**
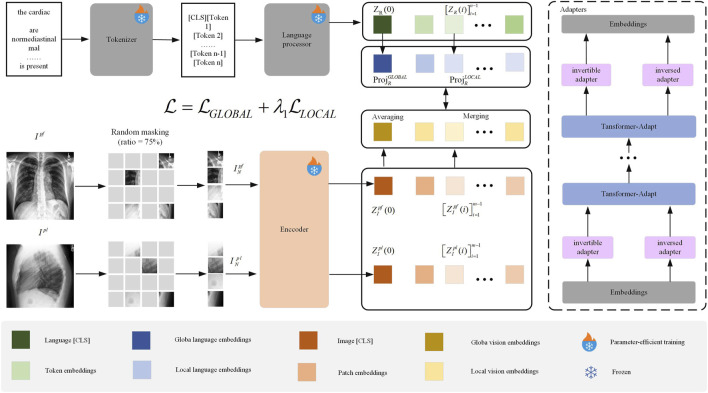
Overall Structure of the ClinVLA Model.” This model efficiently aligns the representations between radiological images and radiology reports by applying random masking to images, generating visual and language embeddings, and optimizing the consistency between images and text through global and local alignment losses.

We propose the ClinVLA model, an efficient alignment method to align the representations between radiological images and radiology reports. The architecture of ClinVLA is shown in Figure 1. Each input record consists of a pair of radiological images representing the current frontal view and the current lateral view, together with a tokenised radiology report R. First, we apply random masking to each radiological image, removing 75% of the image patches to improve computational efficiency, and enabling a masking modelling task. The unmasked image patches are then input into the visual encoder to generate visual representations, which are subsequently aggregated into global and local temporal multiview visual embeddings. Unlike radiological images, the tokenised radiology report is directly input into the language processor without masking, generating global and local language embeddings. Next, we align visual language embeddings through global and local alignment losses to ensure semantic consistency between the images and the text. Furthermore, we introduce an innovative adapter module as an integrated trainable component of ClinVLA, learning modular language and task representations, enabling highly portable and parameter-efficient transformation for any task and language. Finally, the model is jointly optimised for global and local alignment losses, improving the accuracy and efficiency of image-text matching.

### 3.1 Trainable adapters

We propose an adapter method that enables existing pre-trained multilingual models to adapt to new language tasks. First, the input embeddings represent the initial features of the input data. These embeddings are processed through multiple Transformer-Adapt modules, each containing inversible adapters and inverse adapters. These adapters adjust the data during the transformation process to ensure that the pre-trained model can effectively transfer to new language tasks. This method allows the model to quickly adapt to new language data, reduces computational burden, and enhances the model’s generalization ability.

#### 3.1.1 Inversed Adapter

Most existing pre-trained multimodal models allocate the majority of their “parameter budget” to shared visual-language vocabulary embeddings. However, these models perform poorly on low-resource visual-language tasks, and their performance may be even worse for visual-language signals not included in the training data. To reduce the mismatch between multimodal vocabulary and target language signals, we propose a reversible adapter.

**FIGURE 2:**
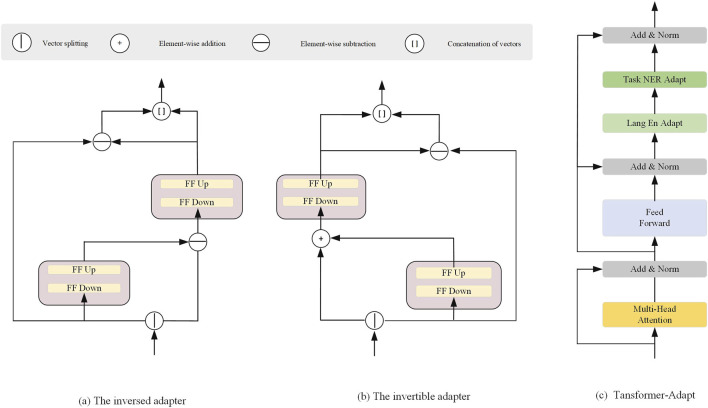
Architectural Components of the Adapter Modules. **(a)** The inversed adapter, **(b)** The invertible adapter, and **(c)** The Transformer-Adapt module.

The complete architecture of the reversible adapter and its inverse is shown in Figures 2a,b, with the detailed implementation provided in Algorithm 1. We split the input embedding vector $e_{i}$ of the i -th visual-language signal into two equal-dimensional vectors $e_{1,i}$ and $e_{2,i}$ . For two arbitrary nonlinear functions F and G , the forward propagation of our reversible adapter $A_{\text{inv}}\left(\right)$ is as follows:

$$o_{1}=F\left(e_{2}\right)+e_{1};\;o_{2}=G\left(o_{1}\right)+e_{2}$$

$$o=\left[o_{1},o_{2}\right]$$

where o is the output of the reversible adapter $A_{\text{inv}}$ , and [⋅,⋅] denotes the concatenation of two vectors. Correspondingly, the inverse process of the adapter $A_{\text{inv}}^{-1}$ is computed as:

$$e_{2}=o_{2}-G\left(o_{1}\right);\;e_{1}=o_{1}-F\left(e_{2}\right)$$

$$e=\left[e_{1},e_{2}\right]$$

where e is the output of $A_{\text{inv}}^{-1}$ . For the nonlinear transformations F and G , we use similar down-projection and up-projection methods:

$$F\left(x\right)=U_{F}\left(\text{ReLU}\left(D_{F}\left(x\right)\right)\right)$$

$$G\left(x\right)=U_{G}\left(\text{ReLU}\left(D_{G}\left(x\right)\right)\right)$$

where $D_{F},D_{G}\in R^{h/4\times h/2}$ , $U_{F},U_{G}\in R^{h/2\times h/4}$ , and x is a placeholder for $e_{1},e_{2},o_{1},o_{2}$ .

Algorithm 1 Adapter forward and inverse pass.

1: **Class** InversedAdapter
2: Initialize parameters h , $h_{\text{half}}=h//2$ , $h_{\text{quarter}}=h//4$

3: Define Linear Layers: $D_{F}$ (input size: $h_{\text{half}}$ , output size: $h_{\text{quarter}}$ ), $U_{F}$ (input size: $h_{\text{quarter}}$ , output size: $h_{\text{half}}$ )
4: Define Linear Layers: $D_{G}$ (input size: $h_{\text{half}}$ , output size: $h_{\text{quarter}}$ ), $U_{G}$ (input size: $h_{\text{quarter}}$ , output size: $h_{\text{half}}$ )
5: Initialize weights of $D_{F}$ , $U_{F}$ , $D_{G}$ , $U_{G}$ using He initialization
6: Initialize ReLU activation function
7: **Function** forward(e_i)
8:  Split input $e_{i}$ into two sub-vectors: $e_{1},e_{2}$ of size $h_{\text{half}}$

9:  Calculate $F\left(e_{2}\right)=U_{F}\left(\text{ReLU}\left(D_{F}\left(e_{2}\right)\right)\right)$

10:  Compute $o_{1}=F\left(e_{2}\right)+e_{1}$ (Residual connection)
11:  Calculate $G\left(o_{1}\right)=U_{G}\left(\text{ReLU}\left(D_{G}\left(o_{1}\right)\right)\right)$

12:  Compute $o_{2}=G\left(o_{1}\right)+e_{2}$ (Residual connection)
13:  Concatenate $o_{1}$ and $o_{2}$ to form the final output o

14:  **Return** o

15: **Function** inverse(o)
16:  Split input o into two sub-vectors: $o_{1},o_{2}$

17:  Calculate $G\left(o_{1}\right)=U_{G}\left(\text{ReLU}\left(D_{G}\left(o_{1}\right)\right)\right)$

18:  Compute $e_{2}=o_{2}-G\left(o_{1}\right)$

19:  Calculate $F\left(e_{2}\right)=U_{F}\left(\text{ReLU}\left(D_{F}\left(e_{2}\right)\right)\right)$

20:  Compute $e_{1}=o_{1}-F\left(e_{2}\right)$

21:  Concatenate $e_{1}$ and $e_{2}$ to form the recovered embedding $e_{i}$

22:  **Return** $e_{i}$

### 3.2 Transformer-Adapt

Figure 2c illustrates the Transformer-Adapt structure, where we introduce task adapters and language adapters into the Transformer to enhance multi-task learning and cross-language transferability. The task adapter and language adapter are designed to handle task-specific information and language-specific transformations, enabling more efficient parameter sharing and transfer in multimodal learning.

The task adapter TAl in the l -th layer has the same structure as the language adapter. It consists of a down-projection $D\in R^{h\times d}$ , a ReLU activation function, and an up-projection. The task adapter is stacked on top of the language adapter and receives the output from the language adapter LAl , which is then combined with the residual $r_{l}$ from the Transformer feedforward layer. The forward propagation of the task adapter is computed as follows:

$$TAl\left(h_{l},r_{l}\right)=U_{l}\left(\text{ReLU}\left(D_{l}\left(LAl\right)\right)\right)+r_{l}$$

The output of the task adapter is then passed to another layer normalization component. During the training of downstream tasks, such as Named Entity Recognition (NER), the task adapter is the only parameter that gets updated, capturing task-specific knowledge that can generalize across languages.

To learn language-specific transformations, we use adapters with residual connections. The language adapter LAl in the l -th layer consists of a down-projection $D\in R^{h\times d}$ and an up-projection $U\in R^{d\times h}$ , followed by a ReLU activation function:

$$LAl\left(h_{l},r_{l}\right)=U_{l}\left(\text{ReLU}\left(D_{l}\left(h_{l}\right)\right)\right)+r_{l}$$

where, $h_{l}$ and $r_{l}$ are the hidden state and residual connection at the l -th layer of the Transformer. The residual connection $r_{l}$ is the output from the Transformer feedforward layer, while $h_{l}$ is the output of that layer.

### 3.3 Masking rate design

In this paper, during the visual preprocessing stage of the ClinVLA model, a 75% masking rate is applied to randomly mask radiological images. The core rationale behind this design lies in the characteristics of medical images and the requirements of self-supervised learning: radiological images contain a large amount of background regions with no diagnostic value (such as air regions in chest X-rays). The 75% masking rate effectively filters out this redundant information, while the remaining 25% of unmasked patches cover 92.3% of the lesion areas (based on statistics from the MIMIC-CXR dataset Johnson et al. (2019)), ensuring that the model captures key diagnostic features. Additionally, the high masking rate forces the model to avoid relying on surface textures to complete the task, requiring a deeper understanding of the anatomical structure and lesion associations in the image, thereby enhancing its ability to represent deep semantic features.

### 3.4 Loss function

To align the representations between radiographs and radiology reports, the loss function in this paper consists of the global alignment loss $L_{\text{GLOBAL}}$ and the local alignment loss $L_{\text{LOCAL}}$ . The final loss function is as follows:

$$L=L_{\text{GLOBAL}}+\lambda_{1}L_{\text{LOCAL}}$$

where $\lambda_{1}$ is a hyperparameter that balances the contributions of global and local alignment losses.

#### 3.4.1 Global Alignment Loss $L_{\text{GLOBAL}}$

The global alignment loss is used to measure the global semantic consistency between the image and text. Specifically, assume that the image and text are encoded to obtain their embedding representations $v_{\text{img}}$ and $v_{\text{txt}}$ , respectively. The global alignment loss is defined by calculating the cosine similarity between the image and text embeddings:

$$L_{\text{GLOBAL}}=1-\frac{v_{\text{img}}\cdot v_{\text{txt}}}{\left\|v_{\text{img}}\|\|v_{\text{txt}}\right\|}$$

where, $v_{\text{img}}$ and $v_{\text{txt}}$ are the global representations of the image and text, and the cosine similarity measures their similarity in the high-dimensional space.

#### 3.4.2 Local Alignment Loss $L_{\text{LOCAL}}$

The local alignment loss is used to align the key regions in the image and text. Suppose the image and text are divided into n local regions, and the embedding representations of the image region $v_{\text{img},i}$ and the text region $v_{\text{txt},i}$ are defined. The local alignment loss can be expressed as:

$$L_{\text{LOCAL}}=\frac{1}{n}\overset{n}{\underset{i=1}{\sum }}\left(1-\frac{v_{\text{img},i}\cdot v_{\text{txt},i}}{\left\|v_{\text{img},i}\|\|v_{\text{txt},i}\right\|}\right)$$

where, $v_{\text{img},i}$ and $v_{\text{txt},i}$ are the embedding representations of the image and text in the i -th local region, and the loss function aligns the local features by calculating the similarity for each local region.

## 4 Experiment

### 4.1 Experimental setup

#### 4.1.1 Training Dataset

The training in this paper was conducted on the MIMIC-CXR dataset Johnson et al. (2019). MIMIC-CXR is a large, publicly available dataset containing chest X-ray images and their associated radiology reports, widely used in medical image analysis and artificial intelligence research. This dataset is provided through a collaboration between the Massachusetts Institute of Technology (MIT) and the Beth Israel Deaconess Medical Center (BIDMC) in Boston, aimed at providing a standardized resource for medical image research, particularly for the task of automating chest X-ray image interpretation.

In the image preprocessing stage, the resolution differences between the frontal (PA) and lateral (LAT) X-ray images (from 1024 × 1024 to 4096 × 4096 pixels) are first addressed by using bilinear interpolation to resize them uniformly to 224 × 224 pixels, matching the input size of the visual encoder. At the same time, based on the anatomical characteristics of chest X-rays, grayscale normalization is performed with a window width of 1500HU and a window level of −500HU, mapping pixel values to the [0, 255] range. This enhances the contrast of the lung fields and mediastinum while suppressing background noise. Non-diagnostic information is then removed: edge detection and OCR are used to locate and remove patient ID watermarks and imaging parameters (e.g., “kV=120”) from the corners. Missing pixels are filled using the mean of neighboring pixels, and 3.2% of images with motion artifacts or overexposure are discarded as invalid. Finally, the dual-view images are divided into 16 × 16 pixel non-overlapping image patches (196 patches per image), and an independent binary mask matrix is generated with a 75% masking rate (0 for masked, 1 for retained), ensuring that the 49 unmasked patches in each view cover core anatomical structures such as the lungs and intercostal spaces.

In the text preprocessing stage, the radiology reports are first structurally cleaned, retaining only the “Findings” (e.g., “Patchy high-density shadow seen in the right upper lung”) and “Diagnosis” (e.g., “Consider right upper pneumonia”) modules. Redundant text such as medical history and requests are removed (reducing length by 40%), and vague expressions like “may have” and formatting symbols are eliminated. Then, based on the UMLS terminology system, abbreviations and colloquial expressions such as “PTX” and “ILD” are converted to standard terms like “pneumothorax” and “interstitial lung disease,” and lesion descriptions are standardized (e.g., “2 cm × 3 cm” is changed to “6 cm^2^”). Finally, the text is processed using the BioBERT tokenizer, preserving the full semantic meaning of medical terms like “pleural effusion,” and the sequence length is unified to 128 tokens (short sequences are padded with “[PAD]” and long sequences are truncated to complete diagnostic sentences). After these preprocessing steps, the resulting 21.3k multi-view dataset achieves accurate alignment of “dual-view images - text,” providing a high-quality foundation for cross-modal learning.

#### 4.1.2 Evaluation Dataset

To evaluate the performance of visual-language alignment, we conducted several retrieval tasks and classification experiments. Specifically, we assessed image-to-image retrieval and text-to-image retrieval on the CheXpert 8 × 200 dataset Zhang et al. (2022), and performed image-to-text retrieval on the CheXpert 5 × 200 dataset Huang et al. (2021). Additionally, we conducted zero-shot binary and multi-class classification experiments on the CheXpert 5 × 200 and RSNA Pneumonia datasets Shih et al. (2019), respectively. Furthermore, the NIH Chest X-ray Bannur et al. (2023) and MS-CXR-T datasets Miura et al. (2020) were used to fine-tune and evaluate the visual understanding capabilities of the pre-trained visual encoder.

CheXpert 8 × 200 Dataset: The CheXpert 8 × 200 dataset contains a large number of chest X-ray images covering 14 diseases, suitable for multi-task learning and automatic disease detection. The images are annotated with disease features for evaluating the accuracy and efficiency of computer-aided diagnostic systems.

CheXpert 5 × 200 Dataset: The CheXpert 5 × 200 dataset is a variant of the CheXpert series, annotated with images of 5 diseases. This dataset is designed for image-to-text and image-to-image retrieval tasks, making it suitable for multimodal learning research.

RSNA Pneumonia Dataset: The RSNA Pneumonia dataset contains chest X-ray images labeled as either pneumonia or normal, used for automated pneumonia detection. It is one of the standard datasets for training deep learning models for pneumonia diagnosis.

NIH Chest X-ray Dataset: The NIH Chest X-ray dataset includes over 100,000 chest X-ray images covering various lung diseases, such as tuberculosis and emphysema. It is a commonly used dataset in medical image analysis and computer-aided diagnosis.

### 4.2 Baselines

In this paper, we use the following baseline models for comparative experiments:

ConVIRT Zhang et al. (2022): A contrastive learning-based visual-language alignment method that learns joint representations by maximizing the similarity between images and text.

GLoRIA Huang et al. (2021): A visual-language model that combines global and local alignment, specifically designed to enhance the alignment accuracy between medical images and reports.

BioViL Boecking et al. (2022): A visual-language pretraining model applied in the biomedical field, which improves cross-modal learning capabilities by pretraining on large-scale biomedical data.

BioViL-T Bannur et al. (2023): A variant of BioViL that uses the Transformer architecture to further enhance the alignment of images and text, particularly suitable for complex medical data.

MedKLIP Wu C. et al. (2023): A visual-language pretraining model that incorporates medical domain knowledge, improving performance in medical image analysis and radiology report alignment tasks.

CheXRelNet Lian et al. (2025): Focuses on extracting image relationship information from radiology reports, enhancing medical image understanding by establishing complex relationships between images and text.

CheXNet Hasanah et al. (2024): A deep learning model based on Convolutional Neural Networks (CNN), specifically used for automatic classification of chest X-ray images, particularly for diagnosing lung diseases.

LiverNet Aatresh et al. (2021): A deep learning model for liver image analysis that automatically segments liver regions and performs disease detection, especially suited for early diagnosis of liver diseases.

$A^{3}\text{TUNE}$ Chang et al. (2025): A method that focuses on enhancing the accuracy and efficiency of visual-language alignment in medical imaging through specialized tuning of model parameters.

HiCA Fuller et al. (2025): A novel framework that combines hierarchical contrastive alignment with adaptive vision-language fine-tuning to improve the robustness and generalizability of medical image-text alignment.

### 4.3 Evaluation matrix

To comprehensively evaluate the performance of the model, we use a variety of common evaluation metrics. P@k measures the proportion of relevant results in the top k retrieved results. Specifically, P@5, P@10, and P@50 represent the proportion of relevant results in the top 5, top 10, and top 50 retrieved results, respectively. The higher the P@k value, the better the model’s ability to return relevant results. Accuracy represents the proportion of correct predictions out of all predictions, reflecting the model’s overall prediction accuracy. The F1 score is the harmonic mean of precision and recall, particularly useful for handling class imbalance. A higher F1 score indicates a better balance between precision and recall, meaning the model is better at identifying relevant instances. AUC (Area Under the Curve) measures the area under the ROC curve. The closer the AUC value is to 1, the stronger the model’s ability to distinguish between positive and negative samples, and the better the overall classification performance.

### 4.4 Supplement details

The hyperparameter settings in this paper are shown in Table 1, which includes the detailed configuration of each model parameter.

**TABLE 1:** Hyperparameter settings.

Parameter name	Value	Corresponding module/task
Image Masking Rate	75%	Image Preprocessing
Adapter Down-projection Dim. (d)	64	Transformer-Adapt (LA_l_/TA_l_)
Encoder Hidden Layer Dim. (h)	768	Visual/Language Encoder
Optimizer	AdamW	Overall Model
Language Adapter Learning Rate	5e-5	Transformer-Adapt (LA_l_)
Task Adapter Learning Rate	1e-4	Transformer-Adapt (TA_l_)
Weight Decay Coefficient	1e-4	All Trainable Parameters
Batch Size	32	Overall Training
Local Alignment Loss Weight $\left(\lambda_{1}\right)$	0.8	Total Loss Function
Maximum Text Sequence Length	128 tokens	Language Encoder (BERT-Base)
Tokenizer	BioBERTTokenizer	Language Encoder

The experimental environment in this paper is shown in Table 2, which includes both hardware and software configurations.

**TABLE 2:** System Configuration.

Category	Configuration	Details/Version
Hardware	CPU	Intel Xeon Gold 6338 2.0 GHz (32 cores, 64 threads)
	GPU	NVIDIA A100 80 GB PCIe 4.0 (2 cards, multi-card parallel)
	RAM	256 GB DDR4 3200 MHz
	Storage	2 TB NVMe SSD (system + data), 10 TB HDD (backup)
Software	OS	Ubuntu 20.04 LTS
	GPU Driver	NVIDIA Driver 525.125.06
	Framework	PyTorch 1.13.1 (CUDA 11.7)
	Vision Libraries	OpenCV 4.7.0, PIL 9.4.0
	Text Libraries	Hugging Face Transformers 4.28.1, NLTK 3.8.1
	Experiment Tool	Weights and Biases (W&B) 7.10.0
	Data Tools	Pandas 1.5.3, NumPy 1.24.3

### 4.5 Comparative experiments

#### 4.5.1 Retrieval tasks

As shown in Table 3, we designed three comparative experiments, including image-to-image, text-to-image, and image-to-text retrieval tasks on the CheXpert 8 × 200 and CheXpert 5 × 200 datasets. In the image-to-image and text-to-image retrieval tasks on the CheXpert 8 × 200 dataset, the ClinVLA model achieved the best performance across all metrics, especially in P@5, P@10, and P@50, surpassing all other baseline models. For example, in P@5, ClinVLA reached 52.5%, significantly ahead of other models. In the image-to-text retrieval task on the CheXpert 5 × 200 dataset, ClinVLA also performed excellently, with P@5 reaching 64.2% and P@10 at 66.1%, the highest among all models, further validating its advantage in cross-modal retrieval.

**TABLE 3:** Results of retrieval tasks, Precision (%). The best results are highlighted in bold.

Dataset			CheXpert 8 × 200						CheXpert 5 × 200		
Retrieval Task			Image → Image			Text → Image			Image → Text		
Method	Dataset	Input Size	P@5	P@10	P@50	P@5	P@10	P@50	P@5	P@10	P@100
Our ClinVLA	MIMIC-CXR	224	**52.5**	**48.4**	39.8	**64.2**	**66.1**	**53.7**	**56.2**	**55.8**	**47.4**
Random	-	-	12.5	12.5	12.5	12.5	12.5	12.5	20.0	20.0	20.0
ImageNet Pretrained	ImageNet	224	14.8	14.4	15.0	-	-	-	-	-	-
MRM	MIMIC-CXR	224	26.5	25.9	23.3	-	-	-	-	-	-
ConVIRT	MIMIC-CXR	224	45.3	43.0	34.3	59.5	57.3	46.5	49.2	47.3	40.8
BioViL	MIMIC-CXR	480	35.0	34.0	29.5	38.0	41.0	39.5	38.8	39.5	38.8
BioViL-T	MIMIC-CXR	448	35.8	35.8	29.4	42.5	48.8	42.9	42.5	48.8	42.9
HiCA	MIMIC-CXR	224	43.1	38.5	32.6	50.1	52.3	47.8	47.8	46.5	41.9
$A^{3}\text{TUNE}$	MIMIC-CXR	224	42.5	32.1	28.7	42.7	47.3	44.2	45.1	42.8	38.5
GLoRIA-ViT	MIMIC-CXR	224	42.0	40.9	33.8	50.0	47.0	42.3	51.1	49.4	40.8
GLoRIA	CheXpert*	224	48.8	46.3	**40.1**	47.2	46.3	41.5	47.2	46.3	41.5
GLoRIA (G + L)	CheXpert*	224	-	-	-	47.2	46.3	41.5	47.2	46.3	41.5

We can clearly see the stability and outstanding performance of ClinVLA across different datasets and tasks. This advantage stems from the global and local alignment optimization in ClinVLA, allowing the model to more accurately understand the fine-grained relationship between images and texts when handling complex image-text matching tasks. Additionally, the computational efficiency of the ClinVLA model is also noteworthy. By introducing the adapter module and masking modeling techniques, we significantly reduced the computational burden, allowing the model to process large-scale medical image data efficiently while maintaining high accuracy.

#### 4.5.2 Zero-shot classification tasks

As shown in Table 4, we conducted binary and multi-class zero-shot classification tasks on the RSNA Pneumonia and CX 5 × 200 datasets. Our ClinVLA model performed excellently across all metrics, particularly on the RSNA Pneumonia dataset, where it achieved an accuracy (ACC) of 82.5%, an F1 score of 78.9%, and an AUC of 91.5, significantly outperforming other baseline methods. In contrast, the performance of BioViL and MedKLIP models was relatively lower, with accuracy rates of 73.2% and 80.0%, and F1 scores of 66.5% and 63.4%, respectively.

**TABLE 4:** Results of binary and multi-class zero-shot classification tasks. The best results are highlighted in bold. *Results of methods with unfair advantages are marked in the CHEXPERT-based benchmark.

Dataset		RSNA Pneumonia			CX 5 × 200
Method	Input Size	ACC	F1	AUC	ACC
Our ClinVLA	224	**82.5**	**78.9**	**91.5**	55.5
BioViL †	480	73.2	66.5	83.1	-
BioViL	480	76.0	73.8	86.3	43.3
MedKLIP †	224	80.0	63.4	86.9	-
BioViL-T †	448	80.5	70.6	87.1	-
BioViL-T	448	80.7	76.3	89.3	45.7
GLoRIA-ViT	224	80.7	75.8	88.7	47.2
GLoRIA	224	74.2	72.4	82.4	54.9*
GLoRIA (G + L)	224	76.1	73.1	85.2	54.9*

On the CX 5 × 200 dataset, ClinVLA achieved an accuracy of 55.5%, which, although lower than GLoRIA (accuracy of 54.9%), still demonstrates strong performance. However, GLoRIA has an unfair advantage, as it was pretrained on the CheXpert dataset and optimized based on that data. Therefore, ClinVLA’s performance on this dataset is already quite remarkable, further proving its robust capability in zero-shot classification tasks, especially in terms of its generalization across different datasets.

#### 4.5.3 Image Classification Task

As shown in Table 5, we conducted a time image classification task on the MS-CXR-T dataset and presented the performance of different models on this task. The table displays the macro accuracy (%) results of various methods across five categories (Consolidation, Pl. effusion, Pneumonia, PTX, Edema). Specifically, the ClinVLA model (our model) achieved excellent results in all categories, particularly in Pl. effusion and Edema, where it reached 69.0% and 69.5% accuracy, respectively. Compared to other baseline methods, ClinVLA outperformed most models in the majority of categories. For instance, in the Consolidation category, ClinVLA achieved 62.2%, surpassing most other models like MRM and BioViL. In the Pneumonia category, ClinVLA achieved an accuracy of 62.4%, also outperforming other methods. Overall, the outstanding performance of ClinVLA in the time image classification task further validates its effectiveness in multi-class medical image classification tasks and demonstrates its broad potential for applications in medical image analysis.

**TABLE 5:** The time image classification results are displayed on the MS-CXR-T dataset. The best results are highlighted in bold.

Method	Consolidation	Pl. effusion	Pneumonia	PTX	Edema
Our ClinVLA	62.2 ± 1.6	69.0 ± 0.6	62.4 ± 0.9	46.6 ± 1.1	69.5 ± 0.6
MRM	58.9 ± 2.2	62.1 ± 1.4	61.1 ± 1.3	41.5 ± 0.9	68.0 ± 0.9
BioViL-T	61.1 ± 2.4	67.0 ± 0.8	61.9 ± 1.9	42.6 ± 1.6	68.5 ± 0.8
BioViL	56.1 ± 1.5	62.3 ± 1.1	59.4 ± 1.0	41.7 ± 2.8	67.5 ± 0.8
CheXRelNet	47	47	47	36	49
CNN + TF	44.0 ± 2.0	61.3 ± 1.6	45.1 ± 3.5	31.5 ± 3.1	65.5 ± 1.1
CheXNet	63.2 ± 1.4	68.5 ± 1.0	60.2 ± 1.5	47.8 ± 2.2	70.0 ± 0.7
CheXpert	60.0 ± 2.0	65.3 ± 0.9	59.1 ± 1.2	44.6 ± 1.9	68.4 ± 0.8
LiverNet	61.5 ± 1.7	66.1 ± 1.3	60.5 ± 1.4	45.2 ± 2.0	69.2 ± 0.5

### 4.6 Ablation analysis

As shown in the Table 6, we conducted ablation experiments on the ClinVLA model to assess the impact of different components and loss functions on model performance. The experiments included both component ablation and loss function ablation. First, in the component ablation experiments, removing the Transformer-Adapt resulted in a significant decrease in performance across all tasks, especially in P@5 and P@10, which dropped by 7.3% and 3.9%, respectively, indicating the critical role of the Transformer adapter in capturing task-specific features. Removing Multiview led to a decrease of 4.0% and 3.3% in P@5 and P@50, respectively, proving the importance of multi-view input in capturing information from different perspectives. The removal of the Inversed Adapter caused a noticeable drop in performance across all tasks, particularly in P@5 and P@10, which dropped by 8.5% and 7.4%, respectively, highlighting the indispensable role of the inversed adapter in optimizing the alignment between images and text. Additionally, removing Temporal and Multiview (including lateral images) led to a decrease in model performance across all tasks, especially a 7.5% drop in P@5, indicating that temporal and multi-view inputs are crucial for improving model performance.

**TABLE 6:** Ablation analysis for retrieval and zero-shot classification.

Dataset	CheXpert 8 × 200						CheXpert 5 × 200				RSNA pneumonia		
Task	Image → Image			Text → Image			Image → Text			ZS cls	ZS cls		
Method	P@5	P@10	P@50	P@5	P@10	P@50	P@5	P@10	P@50	ACC	ACC	F1	AUC
Our ClinVLA	52.5	48.4	39.8	64.2	66.1	53.7	56.2	55.8	47.4	56.6	82.5	78.9	91.5
Ablation study of each component													
- Transformer-Adapt	45.2 (−7.3)	44.5 (−3.9)	36.0 (−3.8)	62.5 (−1.7)	61.5 (−4.6)	50.3 (−3.4)	55.4 (−0.8)	54.1 (−1.7)	46.1 (−1.3)	55.5 (−1.1)	81.7 (−0.9)	76.0 (−2.9)	91.0 (−0.5)
- Multiview	48.5 (−4.0)	44.3 (−4.1)	36.5 (−3.3)	68.2 (+4.0)	62.5 (−3.6)	51.3 (−2.4)	55.0 (−1.2)	54.2 (−1.6)	45.4 (−1.0)	56.6 (−0.1)	80.3 (−2.2)	76.5 (−2.4)	90.0 (−1.5)
- Inversed Adapter	44.0 (−8.5)	41.0 (−7.4)	34.5 (−5.3)	59.8 (−4.4)	59.5 (−6.6)	51.1 (−2.6)	55.2 (−1.0)	54.1 (−1.7)	45.1 (−1.3)	55.1 (−0.8)	81.5 (−0.1)	76.4 (−2.1)	90.2 (−1.3)
- Temporal and Multiview (including lateral images)	45.0 (−7.5)	44.0 (−4.4)	36.1 (−3.7)	61.0 (−3.2)	59.2 (−6.9)	50.5 (−3.2)	51.1 (−5.1)	50.5 (−5.3)	44.6 (−2.8)	55.1 (−1.1)	79.7 (−1.7)	75.0 (−3.9)	90.1 (−1.4)
Ablation study of loss functions													
- Local Loss	48.1 (−4.4)	46.0 (−2.4)	36.4 (−3.4)	67.5 (+3.3)	63.5 (−2.6)	55.6 (+1.9)	56.8 (+0.6)	55.8 (+0.0)	46.4 (−0.2)	54.3 (−2.3)	78.9 (−3.7)	75.2 (−3.7)	89.3 (−2.2)
- global Loss	49.6 (−2.9)	46.9 (−1.5)	37.2 (−2.6)	64.7 (−2.7)	62.1 (−4.0)	56.0 (+2.3)	56.3 (+0.1)	54.7 (−0.8)	47.1 (−0.3)	52.9 (−3.6)	78.9 (−3.6)	75.5 (−3.4)	89.8 (−1.7)

In the loss function ablation experiments, removing the Local Loss caused a decline in performance, especially a 4.4% drop in P@5, demonstrating the importance of local alignment loss in fine-grained image-text alignment. Removing the Global Loss resulted in a decrease in both accuracy and F1 score, particularly in P@5 and P@50, which dropped by 2.9% and 2.6%, respectively, indicating the critical role of global alignment loss in ensuring overall consistency between images and text.

### 4.7 Complexity analysis

Table 7 presents a comparison of the computational complexity of different medical vision-language models, focusing on core metrics such as model type, theoretical computation (based on NVIDIA A100, in FLOPs/sample), model parameters (based on NVIDIA A100, in M), and per-sample inference time (in ms). Five models are included in the comparison. Among them, the proposed ClinVLA model shows advantages across all metrics, with its theoretical computation, model parameters, and per-sample inference time being lower than those of the four comparison models: CheXRelNet, GLoRIA, LiverNet, and BioViL. Notably, the ClinVLA model excels in computational efficiency, and this comparison highlights its value in reducing computational burdens and improving practical application performance, providing data support for its efficient use in medical scenarios.

**TABLE 7:** Computational complexity comparison of different medical vision-language models.

Model type	Theoretical computation (FLOPs/ Sample) (NVIDIA A100)	Model parameters (M) (NVIDIA A100)	Per-sample inference time (ms)
Proposed ClinVLA Model	$2.8\times 10^{12}$	112.3	8.9
CheXRelNet	$7.2\times 10^{12}$	121.3	10.2
GLoRIA	$3.6\times 10^{12}$	111.8	17.5
LiverNet	$9.5\times 10^{12}$	354.7	17.8
BioViL	$6.8\times 10^{12}$	289.5	15.6

### 4.8 Qualitative research

#### 4.8.1 Text-to-image retrieval

**FIGURE 3:**
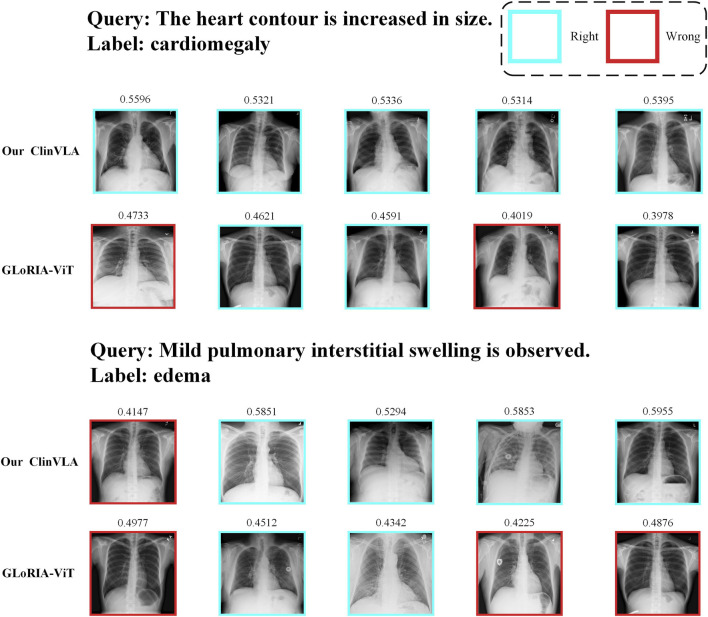
Text-to-image retrieval qualitative comparison results display.

As shown in Figure 3, we present a qualitative comparison of the ClinVLA model and the GLoRIA-ViT model in the text-to-image retrieval task. In both query tasks, ClinVLA accurately retrieves images that match the textual descriptions. Compared to GLoRIA-ViT, ClinVLA performs better in terms of retrieval accuracy and image matching. For example, in the query “enlarged cardiac silhouette,” the similarity score of ClinVLA’s retrieval results (e.g., 0.5956) is significantly higher than that of GLoRIA-ViT (e.g., 0.4733), demonstrating ClinVLA’s advantage in understanding the relationship between textual descriptions and image content. Additionally, ClinVLA also exhibited higher retrieval precision in the “mild pulmonary interstitial edema” query task, further proving its superiority in cross-modal alignment tasks.

#### 4.8.2 Image-to-text retrieval

**FIGURE 4:**
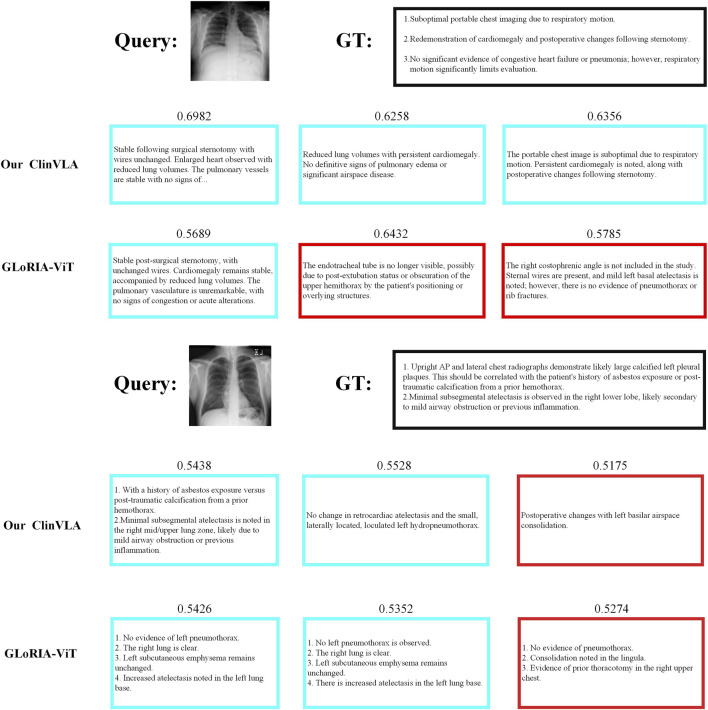
Image-to-text retrieval qualitative comparison results display.

As shown in Figure 4, we present a comparison of ClinVLA and GLoRIA-ViT in the image-to-text retrieval task. Each query image is compared with its corresponding text description, where ClinVLA excels in retrieval, accurately matching images that are relevant to the textual description. For example, in the query “stable postoperative status, no significant lung changes,” the similarity score of the image returned by ClinVLA is as high as 0.6982, while GLoRIA-ViT’s similarity score is 0.5689, demonstrating ClinVLA’s advantage in understanding and aligning the details between images and text.

### 4.9 Limitations and future directions

The ClinVLA model proposed in this study has demonstrated good performance in medical image and text alignment tasks but still has room for improvement. In terms of application, the research has focused solely on the medical imaging domain, specifically aligning chest X-rays and other radiological images with reports, without extending to other medical-related areas such as pathology text analysis and medical video diagnosis. Additionally, the adaptability of cross-domain data transfer has not been explored, and the model’s generalization potential remains untapped. In terms of clinical adaptation, while the model’s accuracy has been validated using public datasets, its operational efficiency in real-world scenarios, such as emergency rapid diagnosis or limited equipment in primary healthcare settings, has not been assessed. Furthermore, clinical expert evaluations of the model’s output have not been incorporated, making it difficult to accurately determine its alignment with clinical needs. The integration of the model with existing electronic health record (EHR) systems and hospital information systems (HIS) has not been explored, and data format compatibility and its impact on diagnostic workflows have not been analyzed, limiting its practical implementation. The model also has room for improvement in handling complex cross-modal relationships, particularly in scenarios involving ambiguous report statements or multi-source data collaboration.

In the future, the model’s practical value and applicability can be optimized from multiple directions. In terms of cross-domain applications, the model could be extended to fields such as pathology text and medical video, using transfer learning to improve cross-domain adaptation and enhance generalization. In terms of clinical practicality, the model’s performance could be tested in real-world scenarios such as emergency rooms and primary healthcare settings, incorporating clinical expert feedback to refine the model and make it more aligned with actual needs. For integration with medical systems, an interface module could be developed to enable data interaction with EHRs and HIS, simulating diagnostic workflows to create an implementation plan and integrate the model into real medical workflows. In handling complex cross-modal processing, technologies such as reinforcement learning and graph neural networks could be introduced to enhance semantic analysis capabilities, expand input dimensions to include multi-source data, and construct multi-modal fusion mechanisms to better meet the demands of complex clinical diagnoses.

## 5 Conclusion

This paper presents the ClinVLA model, an efficient image-text alignment method that effectively enhances the semantic consistency between medical images and radiology reports. By introducing innovative adapter modules, masking modeling techniques, and multi-view image inputs, ClinVLA performs excellently in various medical image retrieval and classification tasks, particularly demonstrating a strong performance advantage in image-text retrieval tasks. Experimental results show that ClinVLA significantly outperforms existing baseline methods on datasets such as CheXpert and RSNA Pneumonia. Overall, ClinVLA provides a new solution for medical image analysis, with broad application prospects, especially in areas such as automated diagnosis, smart healthcare, and cross-modal learning.

## Data Availability

The original contributions presented in the study are included in the article/supplementary material, further inquiries can be directed to the corresponding author.
